# Multi‐Scale Temporal Patterns of Small Cetacean Behaviour in the Bay of Biscay

**DOI:** 10.1002/ece3.74509

**Published:** 2026-10-15

**Authors:** Samuele Tecchio, Laura Couraud, Ludivine Martinez, Achraf Drira, Alessio Maglio

**Affiliations:** ^1^ SINAY Caen France; ^2^ Cohabys La Rochelle University La Rochelle France

**Keywords:** acoustic monitoring, behaviour, dolphin, harbour porpoise, marine mammal, POD

## Abstract

Marine mammals are increasingly exposed to anthropogenic pressures, yet knowledge of their behavioural ecology remains limited, hindering effective conservation planning. Two years of continuous passive acoustic data were collected with porpoise detectors (C‐POD and F‐POD) in the northern Bay of Biscay (northeastern Atlantic Ocean), as part of the baseline assessment for an offshore wind farm development in the South Brittany area. Distinct temporal patterns in small cetacean presence and behaviour were observed. Dolphins exhibited dominant year‐round presence with pronounced peaks in August–September and feeding concentrated during daylight hours. Harbour porpoises ( *Phocoena phocoena* ) showed consistently low activity with autumn‐spring presence and variable diel feeding patterns. Porpoise feeding intensity peaked in November–December, with a diel pattern emerging exclusively at nearshore stations, where buzzes were more frequent during daytime than at night. Environmental modelling identified salinity and current speed as key drivers of dolphin activity, though explained variance remained low, highlighting the complexity of acoustic detection patterns that integrate both occurrence and behaviour. For porpoises, zooplankton biomass was the primary negative driver of activity, while bottom temperature and current speed had positive effects; however, explained variance was even lower than for dolphins, suggesting that factors not captured by the available environmental variables largely govern their distribution. Correlation between C‐POD and F‐POD diel profiles was high for dolphins across all seasons, while it was lower for porpoises. These findings provide a behavioural baseline for this region and directly inform Environmental Impact Assessment procedures by identifying periods of heightened cetacean activity and feeding intensity.

## Graphical abstract

Using porpoise detectors (PODs) deployed continuously over 2 years in the northern Bay of Biscay, we characterised diel, seasonal, and spatial patterns of presence and foraging behaviour in dolphins and harbour porpoises. Dolphin feeding activity tracked the photoperiod year‐round, while porpoise feeding showed a daytime bias restricted to coastal stations and a seasonal peak in autumn–winter. Environmental modelling identified salinity and current speed as key drivers of dolphin activity, and sea surface temperature as the primary negative driver of porpoise activity, though low explained variances highlight the complexity of acoustic detection data.

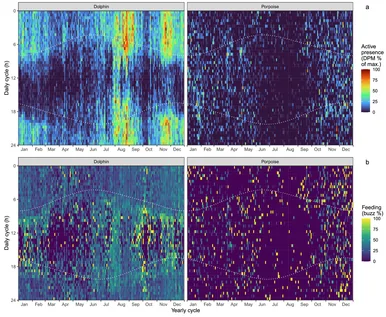

## 1 Introduction

Animals communicate by producing vibrations in their medium, sounds, which carry meaning between individuals (Bradbury and Vehrencamp 2011). The use of sound by animals, and among them marine mammals, is the result of a long adaptive process to an environment not subject to human footprint (Duarte et al. 2021). Studying animal vocalisations allows tapping into their behaviour, giving information about their presence, habitat use, and population ecology (Naguib 2006). Human activities, such as shipping, maritime constructions, or explorations using active sound sources, have significantly increased ocean noise levels over the last century (Erbe et al. 2019; Hildebrand 2009). Such alterations of marine acoustic habitats are recent and occurred relatively fast compared to the evolutionary capacity of animal adaptation, and their consequences are documented at both the individual and population levels (Sørensen et al. 2023; Tyack 2008; Wartzok et al. 2003; Weilgart 2007).

Odontocetes, like other marine mammals, use acoustic signals for all aspects of their active and social life as well as vital functions (Duarte et al. 2021). Sounds produced by odontocetes are mainly of two types: click trains, i.e., short high‐intensity broadband pulses of varying frequency and characteristics, which are produced by all species belonging to this group, and tonal sounds like whistles typical of delphinid species (Au and Hastings 2008; Erbe and Wei 2025). These sounds are associated with different behaviours or activities: clicking can be linked to intra‐specific communication, exploration of the environment, and foraging behaviour (Carlström 2005; Sørensen et al. 2018), while whistles in delphinids are primarily associated with intra‐specific social interactions (Henderson et al. 2012). These characteristics make them easier to detect than with visual techniques. The recent spread of acoustic methods for research and conservation purposes related to the study of cetaceans has opened great opportunities to deepen the knowledge about their ecology, as well as their sensitivity to anthropogenic changes (Castellote et al. 2012; Southall et al. 2016).

Passive Acoustic Monitoring (PAM) has become a standard method to investigate cetacean bioacoustic activity and its changes over time (Bergès et al. 2019). Compared to visual surveys, which are limited to daylight hours and favourable weather, PAM allows continuous long‐term data collection under all conditions. Two broad classes of instruments are commonly employed: continuous broadband hydrophone recorders, which capture the full acoustic spectrum and allow *post hoc* analysis of any sound type, and automated click detectors (PODs), which detect and classify echolocation click trains while discarding raw audio data (Sousa‐Lima et al. 2013). Each approach presents trade‐offs. Broadband recorders offer greater analytical flexibility because they preserve the full acoustic signal, allowing retrospective analyses of multiple vocalisation types. Recent advances in automated detection and machine‐learning classification have further improved their effectiveness for detecting echolocation clicks while retaining the ability to detect whistles and other vocalisations (Fleishman et al. 2023). Automated click detectors, by contrast, offer practical advantages in terms of power autonomy, data storage, and the ability to process large temporal datasets with minimal post‐processing effort. Their suitability relative to broadband recorders has been the subject of debate: performance comparisons between approaches have yielded variable results in the literature (e.g., Roberts and Read 2015; White et al. 2025), and the choice ultimately depends on study objectives, target species, and logistical constraints.

Knowledge of the temporal patterns and environmental drivers of small cetacean distribution in the Bay of Biscay remains incomplete, especially at the fine spatial scale, despite the ecological importance of this region which is demonstrated by the designation of four Important Marine Mammal Areas covering most of the zone (https://www.marinemammalhabitat.org/imma‐eatlas/). The main odontocete species present include the common dolphin ( *Delphinus delphis* ), the bottlenose dolphin ( *Tursiops truncatus* ), the harbour porpoise ( *Phocoena phocoena* ), the striped dolphin ( *Stenella coeruleoalba* ), the Risso's dolphin ( *Grampus griseus* ), and the long‐finned pilot whale ( *Globicephala melas* ) (Gilles et al. 2023; Laran et al. 2017). Although their activity shows a marked seasonality, the details of its drivers remain unclear, as do the precise locations of their preferred habitats and their usage (Lambert et al. 2017). Filling this gap has direct conservation and regulatory relevance: wind farm construction activities are known to impact cetacean hearing thresholds, with both temporary and permanent physiological effects (Carstensen et al. 2006; Dähne et al. 2013; Lucke et al. 2009). Once built, operational wind farms can affect habitat use through indirect effects such as altered prey fields, entanglement risk, or electromagnetic exposure (Bergström et al. 2014; Harris et al. 2025). Under European Union regulations, an offshore wind farm developer must submit an Environmental Impact Assessment (EIA) to permitting authorities. Establishing a pre‐construction baseline of marine mammal activity is therefore both a legal requirement and a scientific necessity. This paper addresses this gap by presenting diel, seasonal, and annual patterns of dolphin and harbour porpoise presence and behaviour in the northern Bay of Biscay, based on 2 years of continuous PAM with PODs as part of the initial state assessment for an offshore wind farm development in Southern Brittany, France.

## 2 Materials and Methods

The study area is located in the Southern Brittany Sea, northern Bay of Biscay, northeastern Atlantic Ocean (Figure 1): this area was designated for the construction of an offshore wind farm and hence subject to an EIA procedure. Small cetacean acoustic activity was monitored using C‐POD and F‐POD devices (Chelonia Ltd., UK), which are autonomous passive acoustic data loggers designed to detect and classify small odontocete echolocation clicks (Ivanchikova and Tregenza 2023). Both C‐POD and F‐POD models were used during the study period. To minimise potential biases linked to differences in detection sensitivity between the two models, devices were rotated between stations and seasons.

**FIGURE 1:**
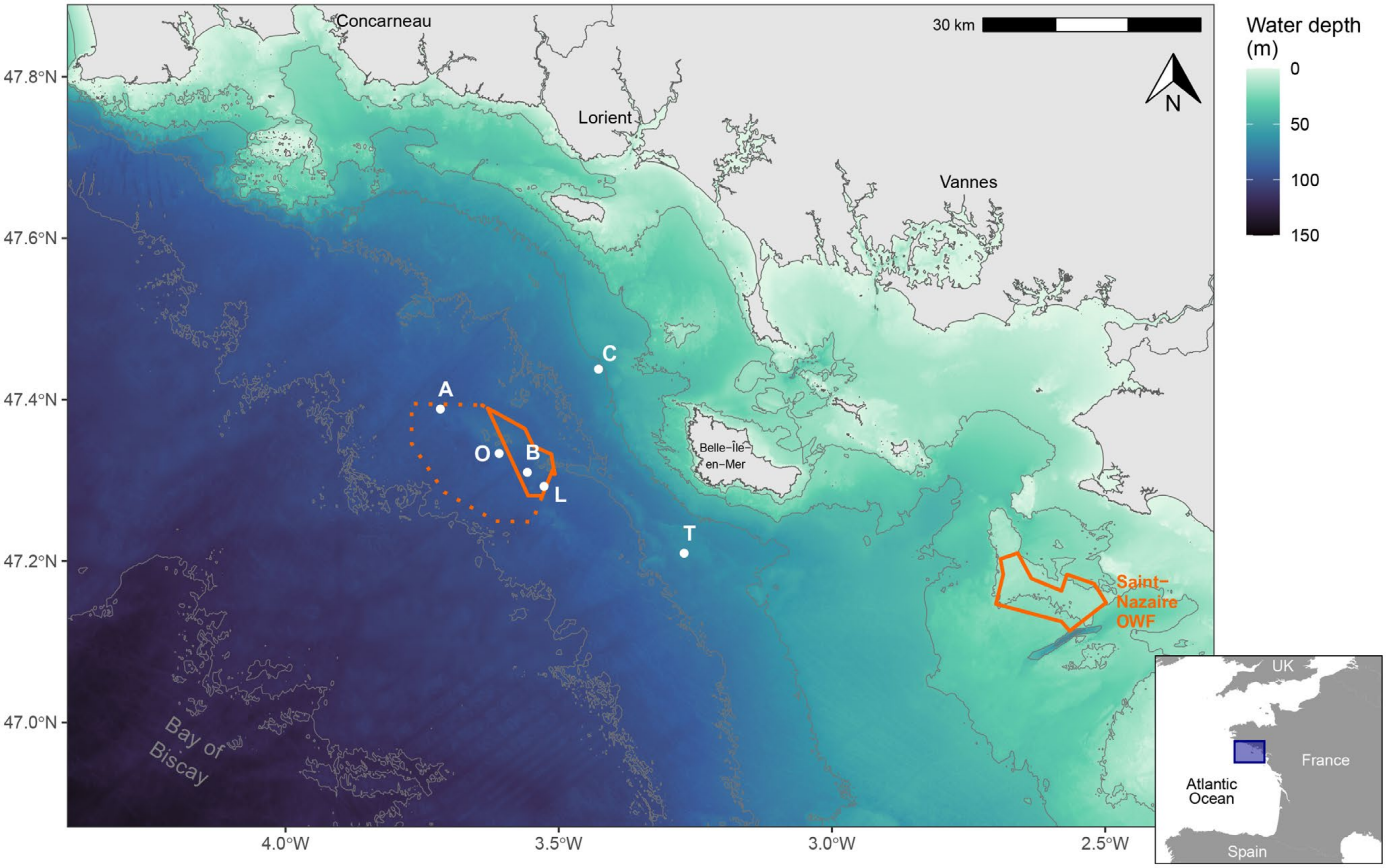
Position of POD deployments in the study area superimposed over a bathymetric colour map. Grey lines indicate 20 m depth contours. The area of the future Southern Brittany wind farm is indicated (solid orange line) as well as its planned extension (dotted orange line). The operational Saint‐Nazaire offshore wind farm (OWF) east of the study area is also shown.

A total of 6 monitoring stations were equipped with PODs and moored at depths of 63–90 m between November 2022 and December 2024 (Figure 2). Four stations (A, O, B, L) were located inside the perimeter of the future offshore wind farm, in a north‐west to south‐east transect. Two stations were located outside the area, one closer to the coast (station C) and the other south of Belle‐Île Island (station T). Devices were mounted on seafloor tripods with hydrophones at a height of 5 m from the bottom and recovered via an acoustically released pick‐up buoy system. After each recovery, data were downloaded from memory cards and verified on board before immediate redeployment.

**FIGURE 2:**
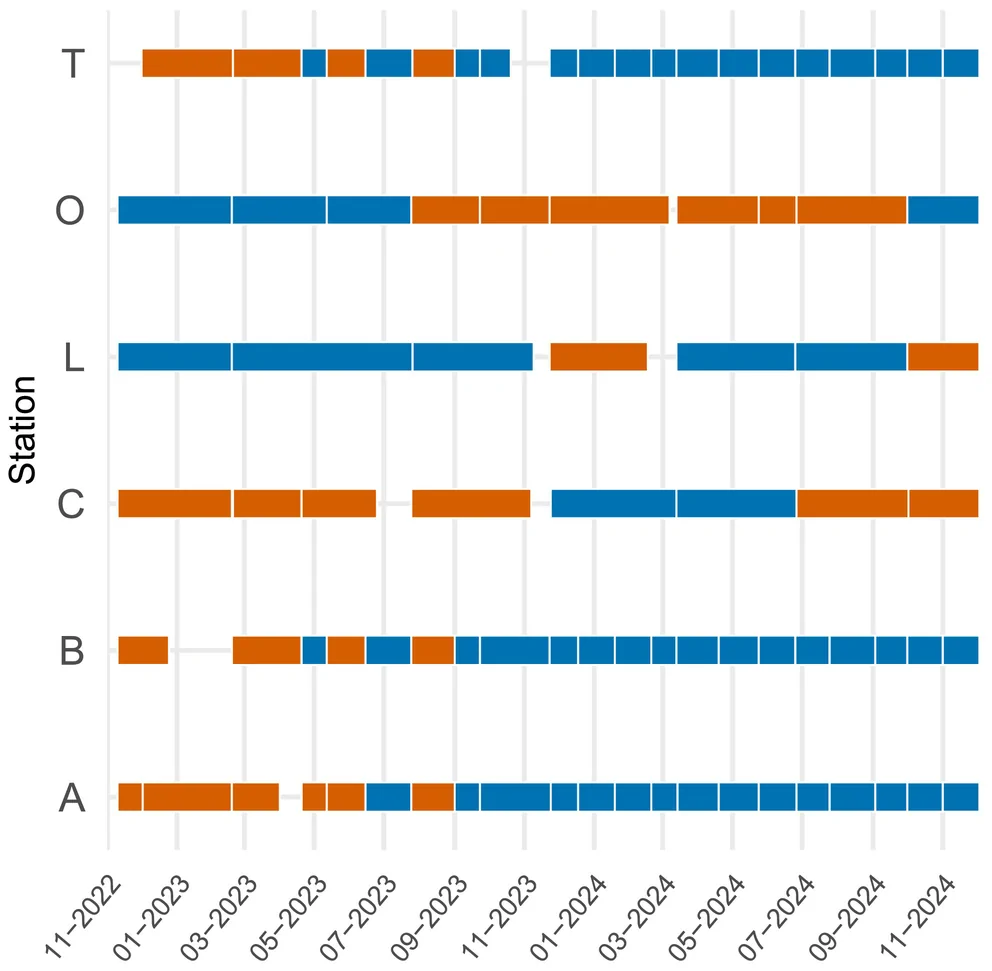
Representation of POD deployments by station over the temporal axis (month‐year). Each deployment ended when data retrieval was performed. Colour coding indicates C‐POD (orange) and F‐POD (blue) deployments.

The proprietary software associated with the PODs was used for the extraction of detections to obtain indicators of the bioacoustics activity of target taxa. The software was set to detect click trains with high probability scores (‘Hi’ mode) as this setting is the most restrictive and lowers the risk of false positives. Detection Positive Minutes (DPM, the number of minutes with at least one click train identified) were obtained from the software for each 10‐min window and attributed to either harbour porpoise ( *Phocoena phocoena* ) or dolphins (Delphinidae spp.). DPM were then aggregated by day for time‐series analysis, hypothesis testing, and environmental modelling, and by 30‐min windows to visualise finer‐scale temporal behaviour. Comparison of means of daily DPM between stations and seasons was performed using non‐parametric Kruskal–Wallis tests followed by Mann–Whitney pairwise tests corrected according to Holm. The significance level was chosen as *α* = 0.05.

To assess whether the diel activity patterns were consistent between device types regardless of their absolute detection rate differences, normalised diel profiles were calculated separately for C‐POD and F‐POD deployments for each species and season. For each combination, the total DPM across all stations and years was summed by 30‐min time bin and normalised to the species‐ and season‐specific maximum (0%–100%). Spearman's rank correlation (*ρ*) was then computed between the C‐POD and F‐POD profiles, limited to those hours where both types recorded at least one detection.

For each click train, the Inter‐Click Interval (ICI) was obtained from the software and used as an indicator of potential feeding behaviour. For both harbour porpoises and dolphins, click trains with ICI ≤ 10 ms were classified as feeding, following references in the literature (Nuuttila et al. 2013; Tellechea 2020). Below this threshold, click trains form “buzzes”, typically emitted in the terminal phase of prey pursuit; therefore, commonly associated with feeding behaviour (Carlström 2005).

The intensity of bioacoustic activity and the intensity of feeding behaviour were analysed on heatmap biplots representing the diel and seasonal cycles. For each combination of day‐of‐year and 30‐min bin, DPM were summed across all stations and all years of the study period. This pooled sum can exceed 30 when multiple stations or years contribute to the same bin. The resulting values were therefore normalised to the species‐specific maximum observed across all bins and expressed as a percentage (0%–100%), providing a relative index of acoustic activity intensity. For the feeding indicator, all click trains recorded within each day‐of‐year × 30‐min bin combination were pooled across stations and years, and the proportion with a mean ICI ≤ 10 ms was expressed as a percentage of the total number of click trains in that bin. The count of trains identified as feeding vs. other trains was tested for independence against the count of trains during the day vs. night by means of Pearson's *χ* ^2^ test.

Several environmental variables were collected to serve as predictors of dolphins' and porpoises' activity for subsequent modelling. Sea surface temperature, bottom temperature, salinity, dissolved oxygen, mean current speed, net primary production, and zooplankton biomass were downloaded from the E.U. Copernicus Marine Data Store, specifically the European North‐West Shelf physics and biogeochemical models. Salinity, dissolved oxygen, current speed, net primary production (modelled as carbon‐equivalent phytoplankton biomass), and zooplankton biomass were obtained by downloading raster data from the study area and calculating the average of each variable for the first 5 m of the water column. Values were then extracted for each station and averaged by day. Tidal ranges (difference between consecutive high and low water levels) were retrieved from the Penfret tidal recording station (Glénan archipelago, 47.72°N, 3.95°W) via the data platform of the French Hydrographic and Oceanographic Service (data.shom.fr). This station is located approximately 40 km from the study area on the same continental shelf margin and within the same macro‐tidal regime. The mean tidal range of the two daily tides was calculated for each day of the study period. Finally, bathymetric data were obtained from the European Marine Observation and Data Network (EMODnet) Digital Terrain Model version 2024.

The daily activity of each species (average daily DPM) was modelled against environmental variables using a zero‐inflated mixture model. This is a two‐part regression approach ideal to model non‐negative count data where there is both overdispersion and an excess number of zeroes (Zeileis et al. 2008). The first component, assuming a binomial distribution with logit link, models the probability of a structural zero, i.e., days with no detected activity vs. false zeroes. The second component assumes a negative‐binomial distribution with log link, to model the actual count against predictor variables, on days where detection is possible. Correlations between predictors were first checked using the Pearson correlation coefficient. High correlations were found between SST and O_2_ (*ρ* = −0.88), salinity and O_2_ (*ρ* = −0.60), and primary productivity and zooplankton (*ρ* = 0.79). Oxygen was therefore excluded from modelling: all other variables were included in the final model to capture potential trophic links with both groups at the base of the food web. The day of the year (an integer between 1 and 365) was also included. To evaluate model performance relative to an alternative approach, a generalised additive model (GAM) with negative binomial errors and smooth terms for the same predictors was also fitted. While the GAM achieved a lower AIC than the zero‐inflated model (Δ_AIC_ = 206 for dolphins, Δ_AIC_ = 72 for porpoises), the zero‐inflated approach was retained because its two‐process structure better aligns with the study's baseline objective, where coefficient interpretability is prioritised over maximum predictive accuracy. An equivalent of *R* ^2^ was estimated with the squared correlation between the model's actual and predicted response: it was used as a descriptive indicator to evaluate the overall performance of each model.

All data processing, statistical analyses, and visualisation were performed using the R language and environment, version 4.5.1 (R Core Team 2025).

## 3 Results

A total of 755 days during which at least one POD recorded data were available, spanning from November 10th, 2022, to December 3rd, 2024, and cumulating 4,508 days across all stations (equal to 6,479,700 cumulative minutes of sensing). Data unavailability was limited and there was no interval during the study period in which no device was recording. The Spearman correlation between C‐POD and F‐POD normalised diel profiles was high for dolphins across all seasons (*ρ* = 0.863–0.922), indicating strong agreement in the timing of diel activity between device types (Figure 3). By contrast, correlations for harbour porpoise were lower and more variable across seasons (*ρ* = 0.257–0.611).

**FIGURE 3:**
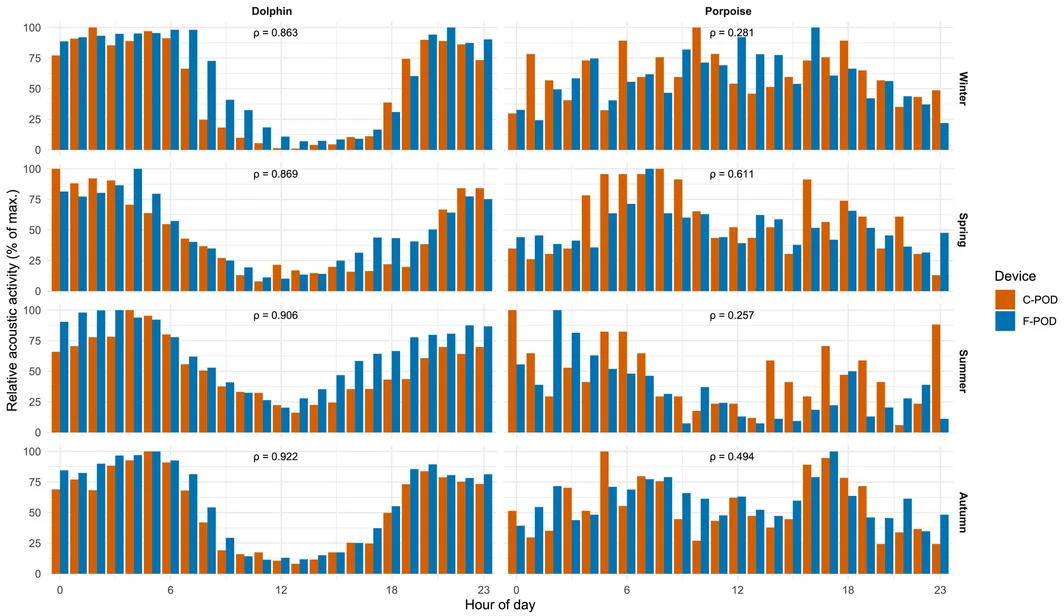
Normalised diel profiles of bioacoustic activity for dolphins (left) and harbour porpoises (right) by season, comparing C‐POD (orange) and F‐POD (blue) deployments. The Spearman's rank correlation (*ρ*) is indicated for each combination of species × season.

### 3.1 Presence

Porpoise clicks were recorded between 33.8% and 51.9% of study days depending on the monitoring station and the proportion of porpoise DPM over the total number of monitored minutes was 0.14%. Dolphin clicks were in contrast recorded between 83.8% and 94.6% of study days, with the most active station being O (only 41 calendar days of silence over 755 recorded), and the proportion of dolphin DPM over the total number of monitored minutes was 5.5%.

Dolphin activity exhibited a marked seasonal cycle (Table 1, Kruskal–Wallis test, *K* = 240, *p* < 0.0001, and Mann–Whitney test *p* < 0.0001 for all pairwise comparisons), and significant spatial structuration (Kruskal–Wallis test, *K* = 409, *p* < 0.0001). Significant minima in spring were found (3–54 DPM d^−1^ across stations), as well as pronounced peaks in late summer (119–241 DPM d^−1^ at A, B, L, O), followed by elevated values into autumn (89–121 DPM d^−1^), and intermediate levels in winter (52–99 DPM d^−1^). The summer peak recurred in both years, concentrated in August–September (Figure 4), and was apparent across stations located within the wind farm project area (A, B, L, O). Station effects for dolphins were dominated by lower DPM at the coastal station C relative to all wind‐farm stations (Mann–Whitney test, *p* < 0.001 for all comparisons) except in winter where levels at C were no different from stations A (*U* = 13,782, *p* = 1) or B (*U* = 12,669, *p* = 0.063). Seasonal differences between A, B, L, and O were mostly non‐significant (0.083 ≤ *p* ≤ 1), except for A in spring and summer vs. L in all seasons (10^−20^ < *p* < 0.004). Station T showed smaller fluctuations than the other stations, with no significant differences at station T across seasons (*p* = 1). Daily DPM at station T were significantly lower than wind‐farm stations A, B and L in autumn (10^−8^ ≤ *p* ≤ 0.0004), and significantly lower than all wind‐farm stations (A, B, L, O) in summer (*p* ≤ 0.0001). T did not differ significantly from station O in autumn (*p* = 1). Values were comparable to all wind‐farm stations in spring (0.092 ≤ *p* ≤ 1), and in winter T did not differ significantly from any station (A, B, C, L or O), despite numerically lower mean DPM relative to L and O (0.356 ≤ *p* ≤ 1).

**TABLE 1:** Detection Positive Minutes (daily DPM, mean ± SD) by station and season, for dolphins and harbour porpoises.

Species	Season	Station					
		A	B	C	L	O	T
Dolphin	Winter	52 ± 59	59 ± 83	66 ± 75	96 ± 115	99 ± 114	58 ± 67
	Spring	33 ± 42	45 ± 51	3 ± 4	54 ± 47	37 ± 44	48 ± 51
	Summer	241 ± 203	185 ± 172	18 ± 28	119 ± 137	150 ± 140	51 ± 69
	Autumn	119 ± 143	119 ± 103	25 ± 36	121 ± 125	89 ± 143	62 ± 76
Porpoise	Winter	1 ± 3	2 ± 5	5 ± 9	3 ± 5	4 ± 7	2 ± 3
	Spring	2 ± 3	2 ± 3	1 ± 3	2 ± 6	2 ± 2	3 ± 5
	Summer	0 ± 2	0 ± 2	1 ± 1	0 ± 1	1 ± 2	1 ± 2
	Autumn	2 ± 4	2 ± 4	5 ± 9	3 ± 5	2 ± 5	4 ± 7

**FIGURE 4:**
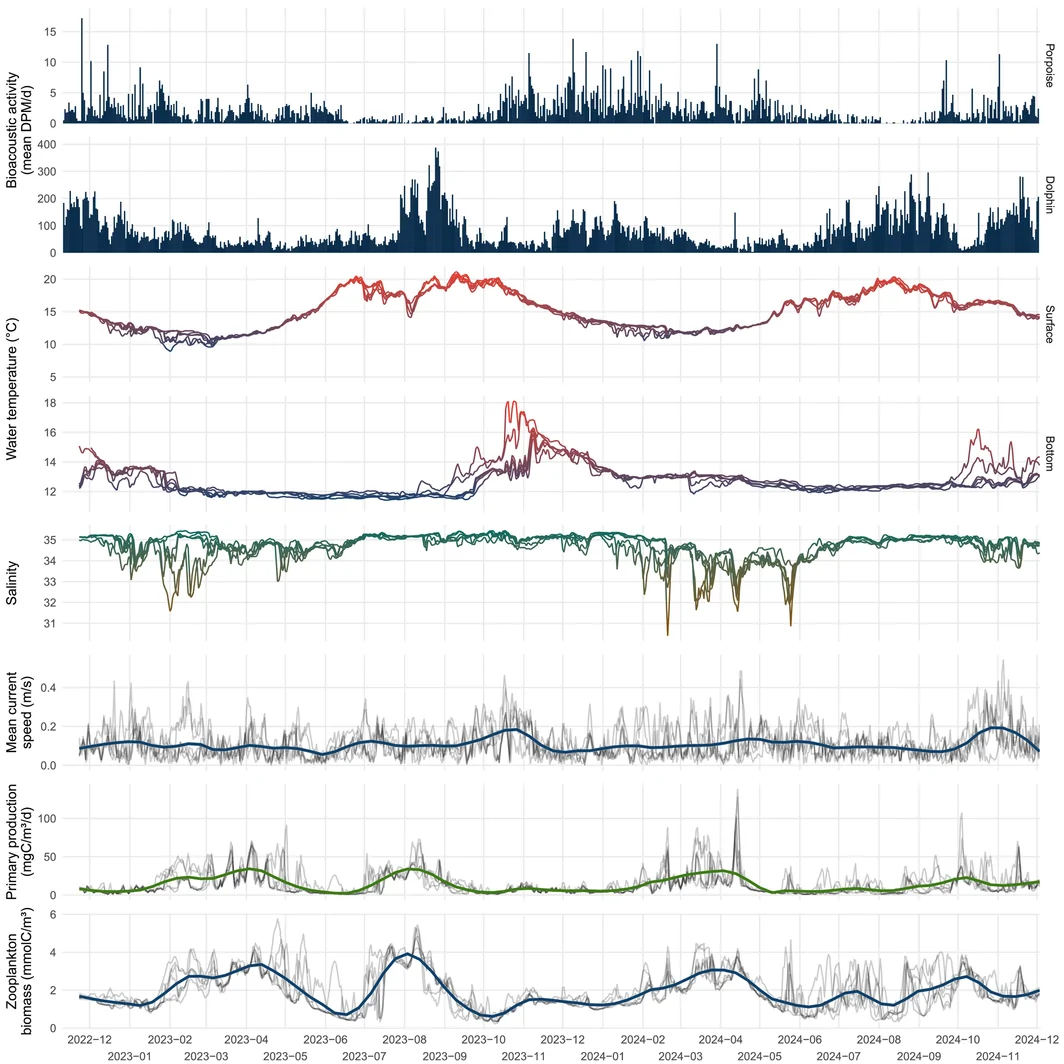
Time series of key variables: Harbour porpoises' and dolphins' mean daily bioacoustic activity in the northern Bay of Biscay (averaging all stations), sea surface temperature (SST), bottom temperature, salinity, mean current speed, primary production, and zooplankton biomass.

Porpoise daily DPM remained low across all strata, typically ≤ 5 daily DPM, with near‐zero activity in summer at most stations. Even with consistently low values, differences were found across stations (Kruskal–Wallis test, *K* = 83, *p* < 0.0001) and seasons (Kruskal–Wallis test, *K* = 298, *p* < 0.0001). Winter and autumn showed the same average DPM across the study period (Mann–Whitney test, *U* = 629,863, *p* = 0.737), as well as winter vs. spring (*U* = 624,892, *p* = 0.128) and spring vs. autumn (*U* = 626,212, *p* = 0.075). Station contrasts for porpoises showed significantly higher DPM at C and T relative to A (*U* = 228,761 and 215,644, *p* < 0.0001 for both), and at T relative to B, L, and O (*U* = 234,221, *p* < 0.0001; *U* = 245,066, *p* < 0.001; *U* = 247,512, *p* = 0.004, respectively). Station C also showed higher DPM than B and L (*U* = 248,354, *p* < 0.0001; *U* = 308,366, *p* = 0.011). Station A had the lowest overall DPM, differing significantly from O and L as well (*U* = 247,220, *p* < 0.0001; *U* = 253,774, *p* = 0.002). C and T did not differ from each other (*U* = 268,361, *p* = 0.754), nor did several mid‐range station pairs (A vs. B, B vs. L, B vs. O, C vs. O, L vs. O; *p* ≥ 0.054). Time‐series averages pooled across stations confirmed consistently low porpoise activity in both years.

### 3.2 Behaviour

The bi‐temporal analysis revealed dolphin and porpoise click emission patterns across temporal gradients (Figure 5). Dolphin bioacoustic activity in the area was more intense during the night (Mann–Whitney test, *U* = 2.8 × 10^9^, *p* < 0.0001), except between August and September as well as in November–December when intense bioacoustic activity was more evenly distributed over the 24 h cycle.

**FIGURE 5:**
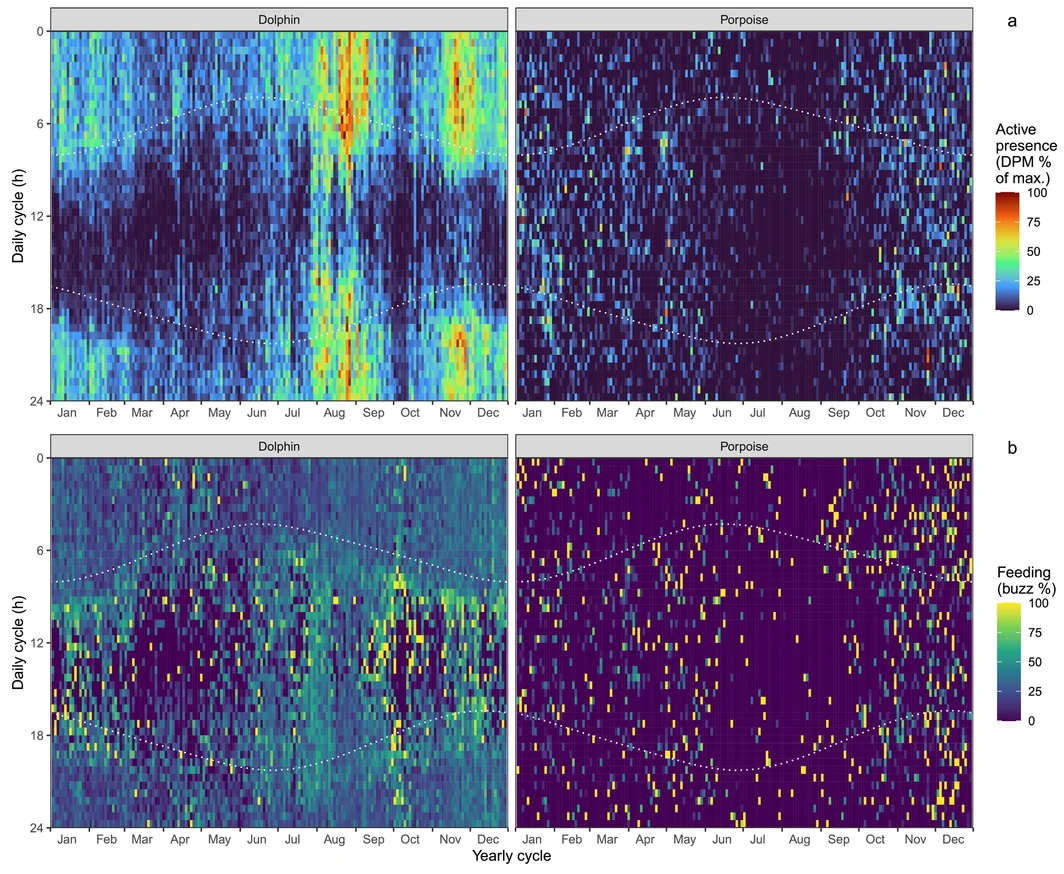
Behaviour of small cetaceans in the study area over daily and yearly cycles, estimated by passive acoustics. (a) Detection Positive Minutes (as percentage of the species maximum), as an indicator of active presence in the area. (b) Proportion of click trains identified as feeding behaviour. Data are averaged for the two study years. Sunrise and sunset times in the region are represented by white dotted lines.

When dolphins are acoustically active, buzz percentage variation follows the photoperiod along the year, with values rising after sunrise and tapering down after sunset. An offset of approximately 1 h is visible between sunrise and the increase in dolphin buzz percentage and between sunset and the decrease of buzz percentage. Photoperiod had a significant effect on dolphins' buzz percentage (*χ* ^2^ = 4,556, *p* < 0.0001) independently of the geographical position. Dolphin buzzes represented 32.0% of their active time during the day, against 25.7% at night.

The bi‐temporal analysis also revealed that 30‐min segments with buzz proportion higher than 75%–80% are mainly found during daylight hours (again offsetting about 1 h after sunrise and sunset). The highest rates of such feeding‐like segments (number of segments per day) in daylight hours are found in the months of October as well as from January to early March. These high rates correspond to low overall bioacoustic activity intensity. Frequent periods of silence, low bioacoustic activity, and low proportion of buzzes are found between March and May in daylight hours.

For porpoises, bioacoustic intensity across temporal gradients showed a reduction in active presence between June and September. Porpoises were present across the daily cycle, and their overall bioacoustic activity did not show differences between day and night (Mann–Whitney test, *U* = 1.5 × 10^9^, *p* = 0.71). No effect of day/night on feeding time was found in the stations located inside the wind farm development area (stations A, O, B, L, *χ* ^2^ = 1.53, *p* = 0.22). Conversely, the effect of day/night is significant for stations closer to the coast (stations C and T, *χ* ^2^ = 23.9, *p* < 0.0001): here, porpoise buzzes represent 20.2% of their active time during the day, and 16.6% during night hours. Feeding‐like segments i.e., high‐buzz‐proportion 30‐min segments, occurred more frequently between November and December.

### 3.3 Environmental Drivers

Sea surface temperatures in the area varied between 8.9°C and 21.1°C (15.2°C ± 2.8°C), following the seasonal cycle. Near‐bottom temperature varied between 11.4°C and 18.1°C (12.7°C ± 0.97°C) and peaked at the end of October 2023. Salinity showed a notable decrease between March and June 2023 when it dropped down to 11.4, otherwise remaining stable at 34.7 ± 0.6. Surface water mass currents had speeds of 0.106 ± 0.071 m s^−1^. Dissolved oxygen fluctuated between 233 and 311 mmol m^−3^ (261.1 ± 14.8 mmol m^−3^). Primary production was 13.9 ± 14.4 mgC m^−3^ d^−1^ averaged over the 2‐year period, reaching its maximum at 138 mgC m^−3^ d^−1^ during the April 2024 bloom. Zooplankton followed the same patterns as primary production, showing a biomass of 1.94 ± 0.99 mmolC m^−3^.

The estimated *R* ^2^ was 0.128 for the model of dolphin bioacoustic activity and 0.058 for the porpoise model, indicating that the chosen environmental variables are only a small part of the predictors of bioacoustic activity in this area (Figure 6). The zero‐inflation part of the models was significant for dolphins (*z* = −15.7, *p* < 0.0001) but not for porpoises (*z* = −0.097, *p* = 0.922). After correction for false negatives, modelling revealed that dolphin activity was increased by depth (*p* < 0.0001), salinity (*p* < 0.0001), current speed (*p* = 0.021), primary production (*p* = 0.042), and day of the year (*p* < 0.0001). Of these variables, salinity was the most influential one (*β* = 0.347), while the least influential was primary production (*β* = 0.005). Dolphin activity was also slightly decreased by bottom temperature (*β* = −0.093, *p* = 0.006).

**FIGURE 6:**
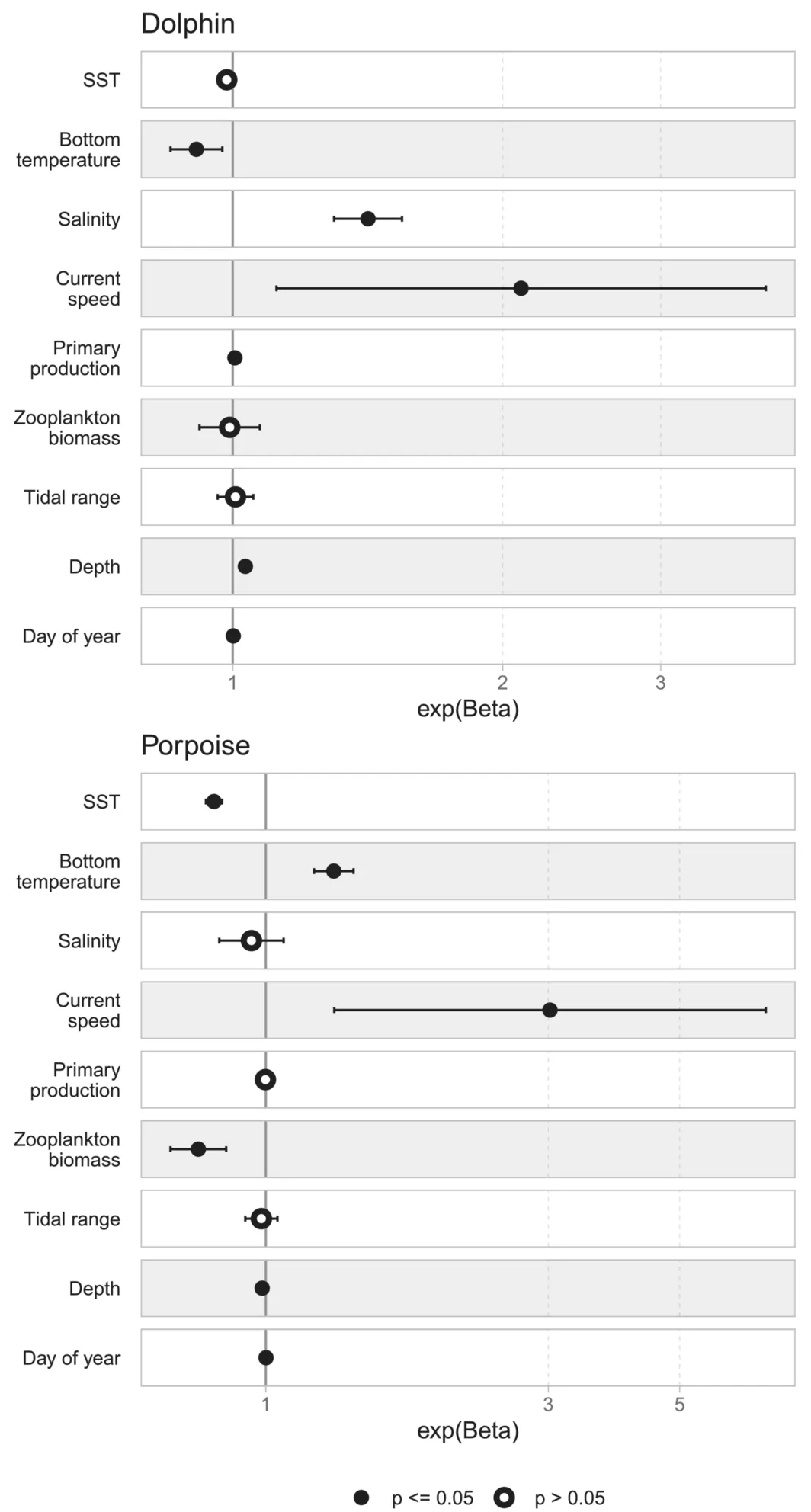
Environmental drivers of dolphin and porpoise bioacoustic activity, as estimated by the zero‐inflated negative binomial models. Values of exp(*β*) higher than 1 indicate a positive relationship, and a negative relationship otherwise.

Porpoise activity was decreased by sea surface temperature (*β* = −0.201, *p* < 0.0001), zooplankton biomass (*β* = −0.262, *p* < 0.0001), and slightly by depth (*β* = −0.014, *p* < 0.0001), while increased by bottom temperature (*β* = 0.264, *p* < 0.0001) and current speed (*β* = 1.105, *p* = 0.009). The day of the year, even if significant, had an almost negligible effect (*β* = 0.0009, *p* = 0.017).

## 4 Discussion

Performance assessment of POD devices against broadband recorders and sightings yielded contrasting results in literature, with authors observing both high and low false‐positive rates for dolphins and porpoises and estimating that C‐PODs generally underreport their presence (Robbins et al. 2016; Roberts and Read 2015; White et al. 2025). Other studies found consistent detections by both C‐PODs and F‐PODs, suggesting their usefulness in multi‐scale temporal studies and over broad applications (Garrod et al. 2018; Ivanchikova and Tregenza 2023). In our case, the consistency of diel patterns between device types differed markedly between the two taxa. For dolphins, normalised diel profiles derived from C‐POD and F‐POD deployments were highly correlated across all seasons. This provides confidence in the conclusions drawn for dolphins despite the unbalanced spatial distribution of device types across stations. For harbour porpoise, however, correlations were lower, consistent with the known sensitivity of this species' acoustic detectability to device performance differences (Todd et al. 2023). However, results here show behavioural structures for both studied species on daily and seasonal scales. Therefore, even considering their limitations, we estimate that PODs produce valuable information on dolphin and porpoise ecology in the study area.

### 4.1 Presence

When studying cetaceans through passive acoustics, it is generally assumed that the detection of acoustic cues from individuals reliably correlates with presence (Fleishman et al. 2023) and hence robust information on their ecology can be derived. Uncertainties about such correlation do exist; for example, in instants with no detections, absence and silent presence cannot be distinguished. Thus, periods of low bioacoustic activity may sometimes not correspond to low presence. Regarding porpoises ( *Phocoena phocoena* ), studies made with acoustic tags show that clicking is almost continuous, albeit with silent periods (about 20 min) which represent 5%–10% of the day (Akamatsu et al. 2007; Linnenschmidt et al. 2013) and may correspond to sleeping behaviour (Wright et al. 2017). It is unknown how these silent periods reflect into potential errors in the analysis of presence variability. However, it can be reasonably assumed that for porpoises, the low duration of silent periods and their small proportion over a day do not affect the assessment of presence variability, especially for long‐term data acquired with continuous recording (100% duty cycle) as in this study.

With regards to dolphins (here referred to species belonging to genus *Stenella*, *Delphinus*, *Tursiops*, *Globicephala*, and *Grampus*, which can be found in the Bay of Biscay and that are supposedly sensed by PODs), the same considerations may apply. Dolphins vocalise frequently, especially because they are highly social species and live in groups. Vocalisations are therefore used not only for foraging, exploring the environment, and navigating, but also for a high proportion for social interactions and intra‐specific communication. However, available studies show that click emissions distributed over 24 h for bottlenose dolphins recorded with acoustic tags may reach 60% of time (Baldachini et al. 2025). Another study on bottlenose dolphins (Roberts 2019) distinguished between proportions reported for foraging or rest behaviour (approximately 85% of recording time clicking) and travelling (57%). Less information exists for other species. Therefore, while the detection of bioacoustic cues reveals dolphin presence and how the area is used by them, the proportion of undetected animals may be higher than for porpoises and hence the ability of distinguishing absence from silent presence may be reduced.

Harbour porpoises were present year‐round in the study area but, compared to other northern European seas, the intensity of overall bioacoustic activity was lower. Along the study period, porpoise detections never exceeded 5 DPM/day on average in any season, while seasonal averages can exceed 250 DPM/day in the North Sea (Calonge et al. 2024; Clausen et al. 2021) as well as in the Irish Sea (Nuuttila et al. 2017). Similar studies for the Bay of Biscay area are scarcer. However, based on available information from the *Iroise Sea* Marine Protected Area (MPA) located approx. 150 km north from the study area, overall detection rates appear comparable to those presented in our study, i.e., lower than other northern European Seas (Torterotot et al. 2025) although a precise comparison is difficult due to different presence indicators used. Estimations from aerial surveys show lower abundances in the Bay of Biscay indeed compared to the English Channel (Gilles et al. 2023) and especially in winter (Laran et al. 2017). Lower bioacoustic activity hence appears to be associated with lower overall abundance. The highest occurrences were recorded at stations C and T, whereas station A showed the lowest activity and the remaining stations occupied an intermediate position. Given the low overall detection rates, these differences likely reflect subtle spatial heterogeneity within the monitored area rather than habitat partitioning.

While studies reporting acoustic occurrences of porpoises in the Bay of Biscay are scant, compared to other northern European areas, they seem to report consistently low values. In other areas such as the North Sea, aerial surveys report overall higher density than the Bay of Biscay (Gilles et al. 2016; Hammond et al. 2013), while studies performed through passive acoustics report both high‐ and low‐density hotspots distributed across the region (Scheidat et al. 2024). This highlights, on one hand, that PAM is a valuable tool to report local differences and effects, and on the other hand, the complementarity with aerial surveys.

Results show seasonal long low‐presence periods, including with no detections, in summer (June to September) and an increased presence from autumn to spring (October to May). These appear consistent with harbour porpoise seasonal variability recorded in the Arcachon Bay, approx. 360 km to the south, where a study showed very low acoustic detections in summer and consistently higher presence the rest of the year (Béesau et al. 2025). In contrast, our results do not compare with the variability observed in the MPA in Brittany mentioned above, despite the proximity to the study area, where presence appears to peak in summer and very low presence or absence is recorded from autumn to spring (Torterotot et al. 2025). Large‐scale aerial survey data from Laran et al. (2017) show that during winter porpoises are mostly found towards coastal areas from north to south of the Bay of Biscay, while the distribution shifts up north and is more widespread towards offshore areas in summer. In this context, the seasonal patterns observed in the present study are consistent with a possible dynamic seasonal shift in habitat use, with porpoises moving northward and offshore in summer and returning southward and inshore in winter. This would place the study area at the northern margin of the latter seasonal range.

Dolphin presence appears dominant compared to porpoises, as expected. Based on available data on community composition of dolphin species, their abundance and habitat preferences in the Bay of Biscay (Gilles et al. 2023; Lambert et al. 2017; Laran et al. 2017), we assume that the vast majority of click detections was due to common dolphins, with a marginal amount of detections potentially due to bottlenose dolphins, Risso's dolphins, pilot whales, and striped dolphins. Our results indicate year‐round dolphin presence in the study area with peaks in summer (exceeding 400 DPM/day on some days, corresponding to more than 25% of detection time over 24 h) as well as during autumn and through winter, although less pronounced. Spring was the season with least recorded dolphin detections, yet 15–30 times higher than harbour porpoises depending on the monitoring station, except for station C closest to the coast where, similarly to harbour porpoise, only few DPM/days were recorded on average in spring (about 0.2%–0.5% of time over 24 h).

The four stations within the future wind‐farm area (A, B, L and O) were located along the same bathymetric contour, and showed comparable seasonal patterns, with an increase in acoustic activity during summer and autumn. This consistency suggests that the monitored wind‐farm footprint represents a relatively homogeneous area with respect to dolphin occurrence. In contrast, station C, located between the coast and the wind‐farm area in shallower waters, exhibited markedly lower dolphin occurrence from spring to autumn, whereas winter occurrence was comparable to the offshore stations. This seasonal pattern suggests changes in dolphin distribution along the coastal‐offshore gradient rather than a permanent site effect. Station T, approximately 18 km east of the future array, showed a different pattern. In this area, dolphin activity remained relatively constant throughout the year and lacked the summer increase observed elsewhere. Consequently, differences between T and the wind‐farm stations were restricted to summer and autumn, suggesting that the seasonal increase in dolphin occurrence was concentrated within the monitored wind‐farm footprint rather than extending uniformly along the offshore transect.

These results may indicate seasonal changes in dolphin distribution, their acoustic behaviour, or both. At a local scale, this is the first time such seasonal changes are reported in the study area. At a larger scale, aerial surveys report stable presence in winter and summer surveys in the Bay of Biscay, though with higher abundance in summer than in winter (Laran et al. 2023) which could explain the difference observed in the present study between detection rates in summer and autumn‐winter. However, the variability of acoustic detections may also be due to changes in acoustic behaviour across seasons instead of population movements in and out.

### 4.2 Behaviour

Acoustic data also revealed behavioural structuring. The findings distinguish between general acoustic presence and feeding‐related buzz activity for both dolphins and harbour porpoises, showing behavioural rhythms influenced by photoperiod, seasonality, and spatial context.

The intensity of overall dolphin bioacoustic activity was significantly higher at night, while feeding‐related buzzes occurred more frequently during daylight hours, with a clear alignment to the photoperiod. The proportion of buzzes increased shortly after sunrise and declined after sunset and were significantly higher during the day. These patterns indicate that exploratory behaviours and communication are dominant at night and that intense foraging behaviour appears temporally separated and focussed on daylight. To our knowledge, this is the first study to document long‐term diel and seasonal variability in dolphin behaviour in the Bay of Biscay using passive acoustics. However, the relationship between feeding behaviour and photoperiod in dolphins reflects known patterns in delphinid ecology (Henderson et al. 2012; Ringelstein et al. 2006; Shane et al. 1986).

Such pattern was evident year‐long, with higher proportions of feeding behaviour detected during daylight including in periods of lower overall bioacoustic activity (spring, early autumn). Large‐scale visual censuses show that dolphins are consistently present in the Bay of Biscay in spring, with offshore species even showing increasing trends in relative abundance between 2004 and 2016 (Authier et al. 2018). The lower bioacoustic activity likely reflects a change in acoustic behaviour or group dynamics rather than a true decrease in abundance. It is possible that lower detection rates reflect a change in group size or a shift towards less social, more silent activities. Consequently, the seasonal peaks in high buzz‐proportion segments observed in October and during spring, despite lower overall acoustic activity, likely reflect periods of focused foraging behaviour by animals that are present but otherwise less vocally active.

Harbour porpoise activity showed a different structure. Overall acoustic presence was reduced in summer months, consistent with previous observations of seasonal shifts in distribution or habitat preference. In contrast to dolphins, porpoises did not exhibit significant diel differences in acoustic activity across the full dataset. However, at stations closer to the coast, feeding buzzes occurred significantly more often during the day, contrasting with nocturnal feeding peaks found at higher latitudes (Schaffeld et al. 2016; Stavenow Jerremalm et al. 2025; Todd et al. 2022), while offshore stations within the wind farm development area showed no diel variation in feeding activity. This spatial divergence suggests that porpoise foraging behaviour is influenced by coastal habitat conditions or prey availability. The lack of diel difference offshore may also reflect the relatively low presence of porpoises in that area, limiting the ability to detect temporal patterns. It is important to interpret such results with caution, as lower detection rates reduce statistical power and increase uncertainty. These results appear though consistent with findings in two areas of the Bay of Biscay—about 150 km north and 360 km south from the study area—which showed weak or no clear diel structuring and feeding activity, distributed evenly between day and night (Stavenow Jerremalm et al. 2025). Seasonal feeding peaks as found in the present study (in winter and spring) seem to compare with the same cited study, especially for the area of Arcachon Bay (south of the Bay of Biscay), confirming previous observations of seasonal shifts in distribution and habitat preference.

While short‐ICI click trains are widely interpreted as indicators of terminal prey capture attempts, they are not uniquely associated with feeding (Arranz et al. 2016). Similar click patterns are reported during socialising and travelling, albeit at proportions of 5% or less (Tellechea 2020). Consequently, the daytime increase observed here in dolphin and nearshore porpoises should be interpreted as an increase in the occurrence of presumed feeding events rather than unequivocal prey capture. In this study we assumed that non‐feeding buzzes occur randomly. If they occur without systematic diel or seasonal patterns, they simply introduce noise. If non‐feeding behaviours producing short ICIs are also more common during daylight, they could contribute to the observed pattern and lead to an overestimation of daytime feeding activity. However, because the same classification criterion was applied consistently throughout the dataset, such uncertainty is unlikely to account entirely for the repeated trends observed. That means that absolute feeding estimates would be inflated, but the relative diel or seasonal patterns remain unchanged, albeit with an increased variance.

### 4.3 Environmental Drivers

The mixture models showed low explanatory power for both dolphins and porpoises. Predictor variables were selected to capture three complementary dimensions of the environment: thermohaline structure and water mass (SST, bottom temperature, salinity), bottom‐up trophic forcing (net primary production and zooplankton biomass), and hydrodynamic processes associated with mixing and prey aggregation (current speed and tidal range). Despite this broad coverage, some structural limitations likely constrain the models' explanatory power.

First, the acoustic metric used (daily DPM) integrates both animal occurrence and behaviour: the relationship between environment and detection probability may therefore mask the relationship between environment and true presence. It is therefore expected that studies based on visual sightings, which more directly record presence, achieve higher explained variances (Becker et al. 2014; Ingrosso et al. 2024). Visual surveys also allow a finer identification of species, while in the present study all Delphinidae were pooled together due to their affinity of acoustic behaviour. This might partially justify the low explanatory power of the model for dolphins. Additionally, visual censuses are representative of short timespans while covering larger areas, frequently identifying geographical factors as influential (e.g., Certain et al. 2008). The positioning of our stations captured a relatively homogenous area in terms of environmental variables, thus providing limited spatial representativity but long‐term temporal dimension. Second, the spatial resolution of the physical and biogeochemical models may fail to resolve mesoscale features (such as thermal fronts, upwellings, and localised prey patches) that might be the real drivers of foraging site selection by cetaceans. Lastly, the six stations were deployed across a relatively homogeneous area in terms of bathymetry and environmental conditions. This provides limited spatial variability across which the environmental models can discriminate, amplifying the relative contribution of unmeasured factors. Visual surveys covering broader areas, including continental shelf edges and offshore gradients, would complement the temporal dimension provided here and help resolve the spatial covariate structure.

For dolphins, the zero‐inflated part was significant, indicating that for count data such as bioacoustic activity, this two‐process modelling framework is appropriate. Within this approach, salinity and current speed were identified as positively impacting delphinids' active presence. Salinity has been identified frequently as a significant predictor of common dolphins' presence in the Atlantic Ocean (Giralt Paradell et al. 2019; Hornsby et al. 2017) as well as in the Mediterranean Sea (Ingrosso et al. 2024). Salinity is rarely a direct driver, but rather a proxy of shifting water masses or fronts, then associated with varying trophic availability. Current dynamics are proxies of water masses to a similar extent. Using fixed stations as in the present study, the identified effects likely reflect fine‐scale variation in local hydrodynamic conditions rather than habitat preferences. As covariates were averaged daily, the models were better suited to detect short‐term physical forcing, such as current dynamics, than indirect trophic drivers. Variables such as primary production and zooplankton biomass may influence cetacean activity with delays, through prey aggregation and transfer across trophic levels. Consequently, the weak contribution of these variables in our models may reflect temporal mismatch and not ecological irrelevance.

Depth was, in this study, an intrinsic characteristic of station positioning (while it is varying on large‐scale surveys). Bathymetry has already been identified as an influential driver of the presence of various species of Delphinidae in studies of the same region (Kiszka et al. 2007) and elsewhere (Cañadas et al. 2002; Gómez de Segura et al. 2008; Hastie et al. 2005), with varying species‐specific responses. Because depth varied little between stations, the ability of the present approach to detect bathymetric effects was limited.

Even if global change factors were less identifiable at this scale, the present study shows that near‐bottom water temperature had a slight negative effect on dolphin presence. Hastie et al. (2005) found a positive relationship of dolphin presence with SST. A two‐season study, in the larger area of the eastern North Atlantic, found a preference of bottlenose dolphin for higher temperatures, especially during winter (Lambert et al. 2017). Preferences for specific surface temperatures were also found for dolphin species in the western North Atlantic Ocean (Chavez‐Rosales et al. 2019). This suggests that behaviours linked with water temperature are observable only at larger spatial scales. Temperature might be a driver of delphinid navigation over long distances rather than exploration when in a confined area.

For harbour porpoises, a clear zero‐inflation was not detected. This likely reflects the extremely low activity of this species in the region, rather than evidence that this stricter modelling approach was less needed. When most data points are close to zero, the statistical power of model‐based inferences is limited. Coupled with the low preference of the region by harbour porpoises, this suggests that strong inferences on environmental influence for *P. phocoena* cannot be drawn from our data alone.

Future modelling efforts could address some of the limitations expressed in this section, by incorporating lagged effects of environmental variables to account for temporal mismatches in trophic propagation, or by integrating acoustic data with concurrent visual surveys. Despite the low explained variances, the models remain informative for the EIA baseline: the identified predictors provide testable hypotheses about habitat use in the study area.

### 4.4 Conclusions

A solid ecological baseline is required for regulatory purposes such as Environmental Impact Assessments (EIA) and/or monitoring programmes related to offshore industrial development. By explicitly integrating environmental drivers and behavioural variations into disturbance analysis, these results will allow distinguishing, during the wind farm construction phase, between whether absence or reduction in detections is attributable to construction activities or to natural factors identified in this study. During construction, knowledge of seasonal drivers and periods of intense feeding activity allows anticipation of periods of heightened sensitivity and adaptation of mitigation measures. As such, the identified activity peaks (August–September for dolphins, autumn‐spring for harbour porpoises) may be considered when planning the most impactful construction activities (e.g., pile driving).

This study also highlights the ecological importance of the northern Bay of Biscay for small cetaceans, especially delphinids. Dolphin detections were recorded during most of the study period and across all stations, indicating regular and sustained use of the area over two consecutive years. The marked seasonal peaks in activity, combined with daylight feeding behaviour, suggest that this sector is not merely crossed occasionally, but is used repeatedly for meaningful activities, including foraging. The future wind farm area and its surroundings form part of an actively used habitat mosaic for delphinids in the Bay of Biscay. Harbour porpoise use was lower, but recurrent detections from autumn to spring indicate that the area is nevertheless part of their seasonal range. The ecological significance of the site therefore differs between taxa and seasons, a distinction that should be considered in future monitoring and mitigation strategies.

This study also emphasises the need for standardisation of acoustic data acquisition and analysis, to enable inter‐site comparisons with other European seas. Complementary work could also explore the integration of visual data to distinguish between abundance variability and behavioural variability.

## Author Contributions

**Samuele Tecchio:** data curation (lead), formal analysis (lead), methodology (equal), visualization (equal), writing – original draft (equal). **Laura Couraud:** formal analysis (equal), investigation (equal), project administration (lead), writing – review and editing (equal). **Ludivine Martinez:** methodology (equal), validation (equal), writing – review and editing (equal). **Achraf Drira:** conceptualization (equal), formal analysis (equal), funding acquisition (equal), writing – review and editing (equal). **Alessio Maglio:** conceptualization (equal), funding acquisition (equal), investigation (equal), methodology (equal), writing – original draft (equal).

## Funding

This work was supported by the French Directorate General for Energy and Climate (DGEC).

## Conflicts of Interest

The authors declare no conflicts of interest.

## Data Availability

The R code and data files that support the findings of this study are available at Figshare (https://doi.org/10.6084/m9.figshare.31850890). Modelling of environmental drivers was conducted using data from the E.U. Copernicus Marine Service Information (https://doi.org/10.48670/moi‐00054 and https://doi.org/10.48670/moi‐00056). The Digital Terrain Model data was obtained from the EMODnet Bathymetry Consortium (https://doi.org/10.12770/cf51df64‐56f9‐4a99‐b1aa‐36b8d7b743a1).
